# An efficient technique for higher order fractional differential equation

**DOI:** 10.1186/s40064-016-1905-2

**Published:** 2016-03-05

**Authors:** Ayyaz Ali, Muhammad Asad Iqbal, Qazi Mahmood UL-Hassan, Jamshad Ahmad, Syed Tauseef Mohyud-Din

**Affiliations:** Department of Mathematics, Faculty of Sciences, HITEC University, Taxila, Pakistan; Department of Mathematics, Faculty of Basic Sciences, University of Wah, Wah Cantonment, Pakistan; Department of Mathematics, Faculty of Sciences, University of Gujrat, Gujrat, Pakistan

**Keywords:** Kawahara equation, Fractional calculus, The $\exp \,\left( { - \varphi \left( \eta \right)} \right)$-expansion method, Traveling wave solutions, Modified Riemann–Liouville derivative

## Abstract

In this study, we establish exact solutions of fractional Kawahara equation by using the idea of $\exp \,\left( { - \varphi \left( \eta \right)} \right)$-expansion method. The results of different studies show that the method is very effective and can be used as an alternative for finding exact solutions of nonlinear evolution equations (NLEEs) in mathematical physics. The solitary wave solutions are expressed by the hyperbolic, trigonometric, exponential and rational functions. Graphical representations along with the numerical data reinforce the efficacy of the used procedure. The specified idea is very effective, expedient for fractional PDEs, and could be extended to other physical problems.

## Background

Most of the scientific problems and phenomena arise nonlinearly in various fields of mathematical physics and applied sciences, such as fluid mechanics, plasma physics, optical fibers, solid-state physics, and geochemistry. The investigation of travelling wave solutions (Shawagfeh 2002; Ray and Bera 2005; Yildirim et al. 2011; Kilbas et al. 2006; He and Li 2010; Momani and Al-Khaled 2005; Odibat and Momani 2007; Abdou 2007; Nassar et al. 2011; Misirli and Gurefe 2011; Noor et al. 2008; Ozis and Koroglu 2008; Wu and He 2007; Yusufoglu 2008; Zhang 2007; Zhu 2007; Wang et al. 2008; Zayed et al. 2004; Sirendaoreji 2004; Ali 2011; Liang et al. 2011; He et al. 2012; Jawad et al. 2010; Zhou et al. 2003; Yıldırım and Kocak 2009; Elbeleze et al. 2013; Matinfar and Saeidy 2010; Ahmad 2014; Bongsoo 2009; Demiray and Pandir 2014, 2015; Lu 2012; Zayed and Amer 2014) of nonlinear evolution equations plays a significant role to look into the internal mechanism of nonlinear physical phenomena. Nonlinear fractional differential equations (FDEs) are a generalization of classical differential equations of integer order. The (FDEs) (Shawagfeh 2002; Ray and Bera 2005; Yildirim et al. 2011; Kilbas et al. 2006) have gained much importance due to exact interpretation of nonlinear phenomena. In recent years, considerable interest in fractional differential equations (He and Li 2010; Momani and Al-Khaled 2005; Odibat and Momani 2007) has been stimulated due to their numerous applications in different fields. However, many effective and powerful methods have been established and improved to study soliton solutions of nonlinear equations, such as extended tanh-function method (Abdou 2007), tanh-function method (Nassar et al. 2011), Exp-function method (Misirli and Gurefe 2011; Noor et al. 2008; Ozis and Koroglu 2008; Wu and He 2007; Yusufoglu 2008; Zhang 2007; Zhu 2007), (*G*^’^/*G*)-expansion method (Wang et al. 2008), homogeneous balance method (Zayed et al. 2004), auxiliary equation method (Sirendaoreji 2004), Jacobi elliptic function method (Ali 2011), Weierstrass elliptic function method (Liang et al. 2011), modified Exp-function method (He et al. 2012), modified simple equation method (Jawad et al. 2010), F-expansion method (Zhou et al. 2003), homotopy perturbation method (Yıldırım and Kocak 2009), Fractional variational iteration method (Elbeleze et al. 2013), homotopy analysis method (Matinfar and Saeidy 2010), Reduced differential transform method (Ahmad 2014), Generalized Kudryashov method for time-fractional differential equations (Demiray and Pandir 2014), The first integral method for some time fractional differential equations(Lu 2012; Zayed and Amer 2014), New solitary wave solutions of Maccari system (Demiray and Pandir 2015), and so on.

In the present paper, we applied the $\exp \,\left( { - \varphi \left( \eta \right)} \right)$-expansion method to construct the appropriate solutions of fractional Kawahara equation and demonstrate the straightforwardness of the method. The fractional derivatives are used in modified Riemann–Liouville sense. The subject matter of this method is that the traveling wave solutions of nonlinear fractional differential equation can be expressed by a polynomial in $\exp \,\left( { - \varphi \left( \eta \right)} \right)$.

1 $$\left( {\varphi^{\prime}\left( \eta \right)} \right) = \exp \left( { - \varphi \left( \eta \right)} \right) + \mu \exp \,\left( {\varphi \left( \eta \right)} \right) + \lambda$$

The article is organized as follows: In “Caputo’s fractional derivative” section, the $\exp \,\left( { - \varphi \left( \eta \right)} \right)$-expansion method is discussed. In “[Sec Sec3]” section, we exert the method to the nonlinear evolution equation pointed out above, in “Solution procedure” section, interpretation and graphical representation of results, and in “Graphical representation of the solutions” section conclusion and references are given.

## Caputo’s fractional derivative

In modelling physical phenomena, using differential equation of fractional order some drawbacks of Riemann–Liouville derivatives were observed In this section we set up the notations and recall some significant possessions.

### **Definition 1**

A real function *f*(*x*), *x* > 0 is said to be in space $C_{\alpha } , \alpha \in \Re ,$ if there exists a real number *p*(>*α*), such that

2 $$f\left( x \right) = x^{p} f_{ 1} \left( x \right), \quad {\text{where}}\;\;f_{ 1} \left( x \right)\, \in C\left[ {0, \infty } \right].$$

### **Definition 2**

A real function *f*(*x*), *x* > 0 is said to be in space $C_{\alpha }^{m} , m \in {\mathbb{N}} \cup \left\{ 0 \right\},$ if *f*^(*m*)^ ∊ *C*_*α*_

### **Definition 3**

Let *f* ∊ *C*_*α*_and $\alpha \ge - 1$, then the (left-sided) Riemann–Liouville integral of order $\mu , \mu > 0$ is given by

3 $$I_{t}^{\mu } f\left( {x,t} \right) = \frac{1}{\varGamma \left( \mu \right)}\mathop \smallint \limits_{0}^{t} \left( {t - {\rm T}} \right)^{\mu - 1} f\left( {x,{\rm T}} \right)d{\rm T} ,\quad t > 0.$$

### **Definition 4**

The (left sided) Caputo partial fractional derivative of *f* with respect to *t*, $f \in C_{ - 1}^{m} , m \in {\mathbb{N}} \cup \left\{ 0 \right\},$ is defined as:

4 $$D_{t}^{\mu } f\left( {x,t} \right) = \frac{{\partial^{m} }}{{\partial t^{m} }}f\left( {x,t} \right), \,\,\mu = m$$

5 $$\;\;\;\;\;\;\;\;\;\;\;\;\;\;\; = I_{t}^{m - \mu } \frac{{\partial^{m} }}{{\partial t^{m} }}f\left( {x,t} \right),\;\;m - 1 \le \mu < m, m \in {\mathbb{N}}$$

Note that

6 $$I_{t}^{\mu } D_{t}^{\mu } f\left( {x,t} \right) = f\left( {x,t} \right) - \mathop \sum \limits_{k = 0}^{m - 1} \frac{{\partial^{k} f}}{{\partial t^{k} }}\left( {x,0} \right)\frac{{t^{k} }}{k!}, \quad m - 1 < \mu \le m, \;m \in {\mathbb{N}}$$

7 $$I_{t}^{\mu } t^{\nu } = \frac{{\varGamma \left( {\nu + 1} \right)}}{{\varGamma \left( {\mu + \nu + 1} \right)}}t^{\mu + \nu } .$$

## Description of $\exp \,\left( { - \varphi \left( \eta \right)} \right)$ expansion method

Now we explain the $\exp \,\left( { - \varphi \left( \eta \right)} \right)$-expansion method for finding traveling wave solutions of nonlinear evolution equations. Let us consider the general nonlinear FPDE of the type

8 $$P\left( {u, u_{t} , u_{x} , u_{xx} , u_{xxx} , \ldots , D_{t}^{\alpha } u, D_{x}^{\alpha } u, D_{xx}^{\alpha } u, \ldots } \right) = 0,\quad 0 \le \alpha \le 1,$$

where $D_{t}^{\alpha } u, D_{x}^{\alpha } u, D_{xx}^{\alpha } u$ are the modified Riemann–Liouville derivatives of *u* with respect to $t, x, xx$ respectively.

Using a transformation $\eta = kx + \frac{{\omega t^{\alpha } }}{\varGamma (1 + \alpha )} + \eta_{0} , k, \omega , \eta_{0}$ are all constants with

9 $$k, \omega \ne 0$$

using the $\exp \,\left( { - \varphi \left( \eta \right)} \right)$-expansion method we have to follow the following steps.

**Step1.** Combining the real variables *x* and *t* by a compound variable *η* we assume

10 $$u\left( {x, t} \right) = u\left( \eta \right),$$

using the traveling wave variable Eqs. (10) and (8) is reduced to the following ODE for *u* = *u*(*η*)

11 $$Q\left( {u,u^{\prime},u^{\prime\prime},u^{\prime\prime\prime},u, \ldots } \right) = 0,$$

where Q is a function of *u*(*η*) and its derivatives, prime denotes derivative with respect to *η*

**Step2.** Suppose the solution of Eq. (11) can be expressed by a polynomial in $\exp \,\left( { - \varphi \left( \eta \right)} \right)$ as follows

12 $$u\left( \eta \right) = a_{n} \left( {{ \exp }\left( { - \varphi \left( \eta \right)} \right)} \right)^{n} + a_{n - 1} \left( {{ \exp }\left( { - \varphi \left( \eta \right)} \right)} \right)^{n - 1} + \cdots ,$$

where $a_{n} , a_{n - 1} , \ldots$ and V are constants to determined later such that *a*_*n*_ ≠ 0 and *φ*(*η*) satisfies equation Eq. (8)

**Step3.** By using the homogenous principal, we can evaluate the value of positive integer *n* between the highest order linear terms and nonlinear terms of the highest order in Eq. (11). Our solutions now depend on the parameters involved in Eq. (1). So Eq. (1) provides the solutions from (13) to (16)

*Case 1λ*^2^ − 4*μ* > 0 and *μ* ≠ 0,

13 $$\varphi \left( \eta \right) = { \ln }\left\{ {\frac{1}{2\mu }\left( { - \sqrt {\lambda^{2} - 4\mu } { \tanh }\left( {\frac{{\sqrt {\lambda^{2} - 4\mu } }}{2}\left( {\eta + c_{1} } \right)} \right) - \lambda } \right)} \right\},$$

where *c*_1_ is a constant of integration.

*Case 2λ*^2^ − 4*μ* < 0 and *μ* ≠ 0,

14 $$\varphi \left( \eta \right) = { \ln }\left\{ {\frac{1}{2\mu }\left( { - \lambda + \sqrt { - \lambda^{2} + 4\mu } { \tan }\left( {\frac{{\sqrt { - \lambda^{2} + 4\mu } }}{2}\left( {\eta + c_{1} } \right)} \right)} \right)} \right\},$$

*Case 3μ* = 0 and $$\lambda \ne 0,$$

15 $$\varphi \left( \eta \right) = - { \ln }\left\{ {\frac{\lambda }{{\exp \left( {\lambda \left( {\eta + c_{1} } \right)} \right) - 1}}} \right\},$$

*Case 4*$$\lambda^{2} - 4\mu = 0, \;\lambda \ne 0,$$ and *μ* ≠ 0,

16 $$\varphi \left( \eta \right) = { \ln }\left\{ {\frac{{2\left( {\lambda \left( {\eta + c_{1} } \right) + 2} \right)}}{{\left( {\lambda^{2} \left( {\eta + c_{1} } \right)} \right)}}} \right\},$$

*Case 5*$$\lambda = 0,$$ and *μ* = 0,

17 $$\varphi \left( \xi \right) = { \ln }\left( {\eta + c_{1} } \right),$$

**Step4.** Substitute Eq. (11) into Eq. (12) and using Eq. (1) the left hand side is converted into a polynomial in $\exp \,\left( { - \varphi \left( \eta \right)} \right)$, equating each coefficient of this polynomial to zero, we obtain a set of algebraic equations for $a_{n} , \ldots \lambda , \mu .$

**Step5.** Eventually solving the algebraic system of equations obtained in step 4 by the use of Maple, we obtain the values of the constants $a_{n} , \ldots ,\lambda$ and *μ*. Substituting *a*_*n*_,… and the general solution of Eq. (8) into solution Eq. (11), we obtain some valuable traveling wave solutions of Eq. (8).

## Solution procedure

Consider the generalized form of fractional order nonlinear Kawahara equation.

18 $$D_{t}^{\alpha } u + \beta uu_{x} + \alpha u_{xxx} - \delta u_{xxxxx} = 0, \quad 0 < \alpha \le 1$$

where $\alpha , \beta$ and *δ* are some nonzero parameters, taking $\alpha = 1, \beta = 1$ and $\delta = - 1$, the model equation is given as. We can convert equation Eq. (18) into an ordinary differential equation.

19 $$- Vu^{\prime} + uu^{\prime} + u^{\prime\prime\prime} - u^{\prime\prime\prime\prime} = 0,$$

where the prime denotes the derivative with respect to *η*. Now integrating equation Eq. (19), we have,

20 $$- Vu + \frac{1}{2} u^{2} + u^{\prime\prime} - u^{\prime\prime\prime\prime} + C = 0,$$

Balancing the $u^{\prime\prime\prime\prime}$ and *u*^2^ by using homogenous principal, we have

$$\begin{aligned} 2M &= M + 4, \hfill \\ M &= 4. \hfill \\ \end{aligned}$$

Then the trial solution of equation Eq. (19) can be expressed as follows,

21 $$u\left( \eta \right) = a_{0} + a_{1} \left( {{ \exp }\left( { - \varphi \left( \eta \right)} \right)} \right) + a_{2} \left( {{ \exp }\left( { - \varphi \left( \eta \right)} \right)} \right)^{2} + a_{3} \left( {{ \exp }\left( { - \varphi \left( \eta \right)} \right)} \right)^{3} + a_{4} \left( {{ \exp }\left( { - \varphi \left( \eta \right)} \right)} \right)^{4} ,$$

where $a_{ 4} \ne 0, \;a_{0} , a_{1} ,a_{2}$ and *a*_3_ are constants to determined, while *λ*, *μ* are arbitrary constants.

Substituting $u, u^{\prime}, u^{\prime\prime}, u^{\prime\prime\prime}, u^{\prime\prime\prime\prime} , u^{2}$ into Eq. (20) and then equating the coefficients of $\exp \,\left( { - \varphi \left( \eta \right)} \right)$ to zero, we get the set of algebraic equations, we obtain the following solution.

### **Solution 1**

22 $$\left\{ \begin{gathered} V = 1680\mu ^{2} - \frac{{36}}{{169}} - a_{0} ,a_{0} = a_{0} ,a_{1} = - \frac{{3360}}{{13}}\mu ^{2} \sqrt {676\mu + 13} ,\;a_{2} = \frac{{1680}}{{13}}\left( {1 + 78\mu } \right)\mu ^{2} ,\;\lambda = - \frac{1}{{13}}\sqrt {676\mu + 13} , \hfill \\ a_{3} = - \frac{{3360}}{{13}}\mu ^{3} \sqrt {676\mu + 13} ,\;a_{4} = 1680\mu ^{4} ,\;C = - \frac{{60480}}{{169}}\mu ^{2} + 1411200\mu ^{4} - 1680\mu ^{2} a_{0} + \frac{{36}}{{169}}a_{0} + \frac{1}{2} + a_{0}^{2} , \hfill \\ \end{gathered} \right.$$

where *λ* and *μ* are arbitrary constants.

Now substituting the values into Eq. (21), we obtain,

23 $$u\left( \eta \right) = a_{0} + - \frac{3360}{13}\mu^{2} \sqrt {676\mu + 13} \left( {{ \exp }\left( { - \varphi \left( \eta \right)} \right)} \right) + \frac{1680}{13}\left( {1 + 78\mu } \right)\mu^{2} \left( {{ \exp }\left( { - \varphi \left( \eta \right)} \right)} \right)^{2} - \frac{3360}{13}\mu^{3} \sqrt {676\mu + 13} \left( {{ \exp }\left( { - \varphi \left( \eta \right)} \right)} \right)^{3} + 1680\mu^{4} ,$$

Now substituting Eq. (13), (14), (15), (16) and (17) into Eq. (23) respectively, we get the following five traveling wave solutions of the Kawahara equation.

*Case 1* When $\lambda^{ 2} - 4\mu > 0$ and *μ* ≠ 0, we obtain the hyperbolic function traveling wave solution.

24 $$\begin{aligned} u_{1} \left( \eta \right) &= a_{0} - 1680\mu^{2} + \frac{105}{169} \\&- \frac{210}{169}tanh\left( {\frac{1}{4394}\left( {169x + \frac{{283920t^{\alpha } \mu^{2} }}{{\varGamma \left( {\alpha + 1} \right)}} - \frac{{36t^{\alpha } }}{{\varGamma \left( {\alpha + 1} \right)}} - \frac{{169t^{\alpha } a_{0} }}{{\varGamma \left( {\alpha + 1} \right)}}} \right)\sqrt {13} } \right)^{2} \\&+ \frac{105}{169}tanh\left( {\frac{1}{4394}\left( {\begin{array}{*{20}c} {169x + \frac{{283920t^{\alpha } \mu^{2} }}{{\varGamma \left( {\alpha + 1} \right)}}} \\ { - \frac{{36t^{\alpha } }}{{\varGamma \left( {\alpha + 1} \right)}} - \frac{{169t^{\alpha } a_{0} }}{{\varGamma \left( {\alpha + 1} \right)}}} \\ \end{array} } \right)\sqrt {13} } \right)^{4} , \end{aligned}$$

*Case 2* When *λ*^2^ − 4*μ* < 0 and *μ* ≠ 0, we obtain trigonometric solution

25 $$\begin{aligned} u_{2} \left( \eta \right) = a_{0} - 1680\mu^{2} + \frac{105}{169} \\&- \frac{210}{169}tanh\left( {\frac{1}{4394}\left( {169x + \frac{{283920t^{\alpha } \mu^{2} }}{{\varGamma \left( {\alpha + 1} \right)}} - \frac{{36t^{\alpha } }}{{\varGamma \left( {\alpha + 1} \right)}} - \frac{{169t^{\alpha } a_{0} }}{{\varGamma \left( {\alpha + 1} \right)}}} \right)\sqrt {13} } \right)^{2} \\&+ \frac{105}{169}tanh\left( {\frac{1}{4394}\left( {\begin{array}{*{20}c} {169x} \\ { + \frac{{283920t^{\alpha } \mu^{2} }}{{\varGamma \left( {\alpha + 1} \right)}}} \\ { - \frac{{36t^{\alpha } }}{{\varGamma \left( {\alpha + 1} \right)}}} \\ { - \frac{{169t^{\alpha } a_{0} }}{{\varGamma \left( {\alpha + 1} \right)}}} \\ \end{array} } \right)\sqrt {13} } \right)^{4} , \end{aligned}$$

*Case 3* When *μ* = 0 and $\lambda \ne 0,$ we obtain exponential solution.

26 $$u_{3} \left( \eta \right) = 1/\left( {52\mu + 1} \right)^{2} \left[ {a_{0} + 1135680\mu^{4} e^{{\frac{ - 1}{2197}\sqrt {676\mu + 13} \left( {169x + \frac{{283920t^{\alpha } \mu^{2} }}{{\varGamma \left( {\alpha + 1} \right)}} - \frac{{36t^{\alpha } }}{{\varGamma \left( {\alpha + 1} \right)}} - \frac{{169t^{\alpha } a_{0} }}{{\varGamma \left( {\alpha + 1} \right)}}} \right) }} + 43680\mu^{3} e^{{\frac{ - 1}{2197}\sqrt {676\mu + 13} \left( {169x + \frac{{283920t^{\alpha } \mu^{2} }}{{\varGamma \left( {\alpha + 1} \right)}} - \frac{{36t^{\alpha } }}{{\varGamma \left( {\alpha + 1} \right)}} - \frac{{169t^{\alpha } a_{0} }}{{\varGamma \left( {\alpha + 1} \right)}}} \right) }} + 1680\mu^{2} e^{{\frac{ - 2}{2197}\sqrt {676\mu + 13} \left( {169x + \frac{{283920t^{\alpha } \mu^{2} }}{{\varGamma \left( {\alpha + 1} \right)}} - \frac{{36t^{\alpha } }}{{\varGamma \left( {\alpha + 1} \right)}} - \frac{{169t^{\alpha } a_{0} }}{{\varGamma \left( {\alpha + 1} \right)}}} \right) }} + 1703520\mu^{4} e^{{\frac{ - 2}{2197}\sqrt {676\mu + 13} \left( {169x + \frac{{283920t^{\alpha } \mu^{2} }}{{\varGamma \left( {\alpha + 1} \right)}} - \frac{{36t^{\alpha } }}{{\varGamma \left( {\alpha + 1} \right)}} - \frac{{169t^{\alpha } a_{0} }}{{\varGamma \left( {\alpha + 1} \right)}}} \right) }} + \cdots 87360\mu^{3} e^{{\frac{ - 2}{2197}\sqrt {676\mu + 13} \left( {169x + \frac{{283920t^{\alpha } \mu^{2} }}{{\varGamma \left( {\alpha + 1} \right)}} - \frac{{36t^{\alpha } }}{{\varGamma \left( {\alpha + 1} \right)}} - \frac{{169t^{\alpha } a_{0} }}{{\varGamma \left( {\alpha + 1} \right)}}} \right) }} - 4258800\mu^{4} - 174720\mu^{3} } \right],$$

*Case 4* When $\lambda^{2} - 4\mu = 0, \;\lambda \ne 0,$ and *μ* ≠ 0, we obtain rational function solution.

27 $$u_{4} \left( \eta \right) = \frac{{ - \left[ {\begin{array}{*{20}l} { - \frac{{84464623357806182400 \left( {t^{\alpha } } \right)^{3} \mu^{6} }}{{\varGamma \left( {\alpha + 1} \right)^{3} }}} \\ { + \frac{{14434947944202240 \left( {t^{\alpha } } \right)^{3} \mu^{4} }}{{\varGamma \left( {\alpha + 1} \right)^{3} }}} \\ { + \cdots - \frac{{71262235786560 \sqrt {676\mu + 13} a_{0}^{2} \left( {t^{\alpha } } \right)^{2} \mu^{2} }}{{\varGamma \left( {\alpha + 1} \right)^{2} }}} \\ \end{array} } \right]}}{{\left[ {\sqrt {676\mu + 13} \left( {\begin{array}{*{20}l} {169x + \frac{{283920t^{\alpha } \mu^{2} }}{{\varGamma \left( {\alpha + 1} \right)}}} \\ { - \frac{{36t^{\alpha } }}{{\varGamma \left( {\alpha + 1} \right)}} - \frac{{169t^{\alpha } a_{0} }}{{\varGamma \left( {\alpha + 1} \right)}}} \\ \end{array} } \right)\left( {52\mu + 1} \right)^{4} } \right]}},$$

*Case 5* when $\lambda = 0,$ and *μ* = 0, we obtain rational function solution.

28 $$u_{5} \left( \eta \right) = - \frac{{20160\mu^{3} xt^{\alpha } a_{0} }}{{\varGamma \left( {\alpha + 1} \right)}}{-}\frac{3360}{13} \frac{{\mu^{2} xt^{\alpha } a_{0} }}{{\varGamma \left( {\alpha + 1} \right)}} - \frac{26127360}{28561}\frac{{\left( {t^{\alpha } } \right)^{3} a_{0} \mu^{4} x}}{{\varGamma \left( {\alpha + 1} \right)^{3} }} + \cdots \frac{3360}{13}\mu^{2} \sqrt {676\mu + 13} x$$

### **Solution 2**

29 $$\left\{ \begin{gathered} V = 1680\mu ^{2} - \frac{{36}}{{169}} - a_{0} ,a_{0} = a_{0} ,\;a_{1} = \frac{{3360}}{{13}}\mu \sqrt {676\mu + 13} ,\;a_{2} = \frac{{1680}}{{13}} + 10080\mu ,\;\lambda = \frac{1}{{13}}\sqrt {676\mu + 13} , \hfill \\ a_{3} = \frac{{3360}}{{13}}\sqrt {676\mu + 13} ,a_{4} = 1680,\;C = - \frac{{60480}}{{169}}\mu ^{2} + 1411200\mu ^{4} - 1680\mu ^{2} a_{0} + \frac{{36}}{{169}}a_{0} + \frac{1}{2}a_{0}^{2} \hfill \\ \end{gathered} \right.,$$

### **Solution 3**

30 $$\left\{ \begin{gathered} V = 1680\mu ^{2} - \frac{{36}}{{169}} - a_{0} ,a_{0} = a_{0} ,\;a_{1} = - \frac{{3360}}{{13}}\mu \sqrt {676\mu + 13} ,\;a_{2} = \frac{{1680}}{{13}} + 10080\mu ,\;\lambda = - \frac{1}{{13}}\sqrt {676\mu + 13} , \hfill \\ a_{3} = - \frac{{3360}}{{13}}\sqrt {676\mu + 13} ,\;a_{4} = 1680,\;C = - \frac{{60480}}{{169}}\mu ^{2} + 1411200\mu ^{4} - 1680\mu ^{2} a_{0} + \frac{{36}}{{169}}a_{0} + \frac{1}{2}a_{0}^{2} \hfill \\ \end{gathered} \right.,$$

### **Solution 4**

31 $$\left\{ {\begin{array}{*{20}l} {V = 560\mu^{2} - \frac{280}{13}\mu - a_{0} + \frac{4371}{33800} - \frac{483}{33800}\iota \sqrt {31} + \frac{14}{845}\mu \left( { - 2015 + 67600\mu + 195\iota \sqrt {31} } \right), \ldots , } \hfill \\ {a_{0} = a_{0} , \;a_{4} = 1680,} \hfill \\ \end{array} } \right.,$$

Similarly, we can find the other exact solution of remaining solutions, while one solution is analyzed.

### Graphical representation of the solutions

The graphical illustrations of the solutions are given below in the figures with the aid of Maple.

## Physical interpretation

**Fig. 1:**
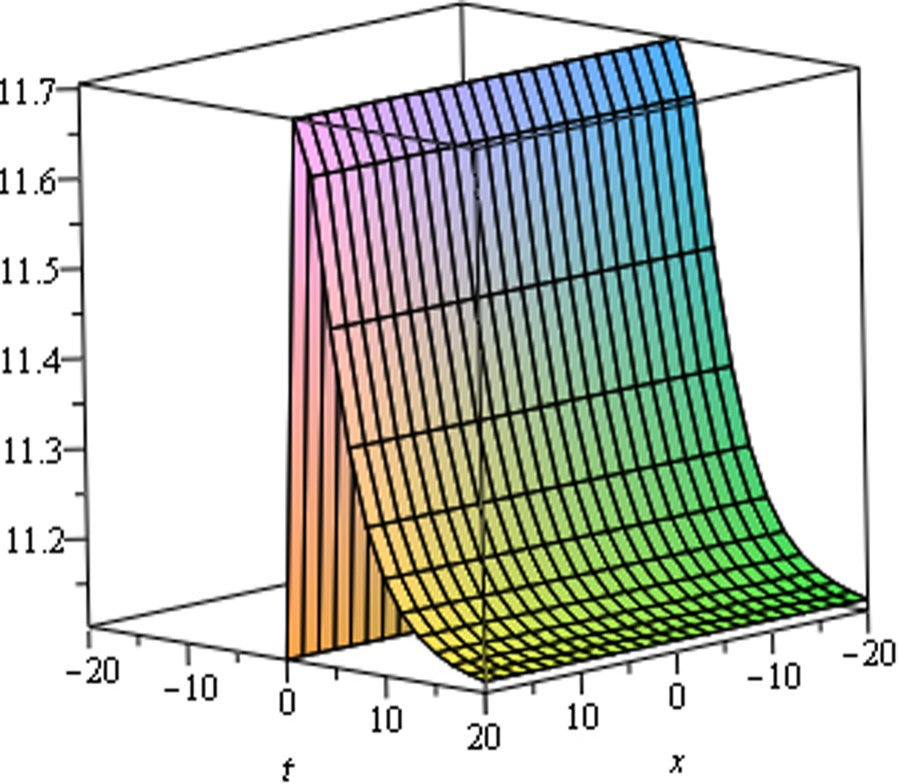
Singular solitary wave solution $u_1 \left({\eta}\right)$ when ${\text{a}}_{0} = 11.1, \mu = - 0.0002, {\text{x}} = 15, \alpha = 0.25$

**Fig. 2:**
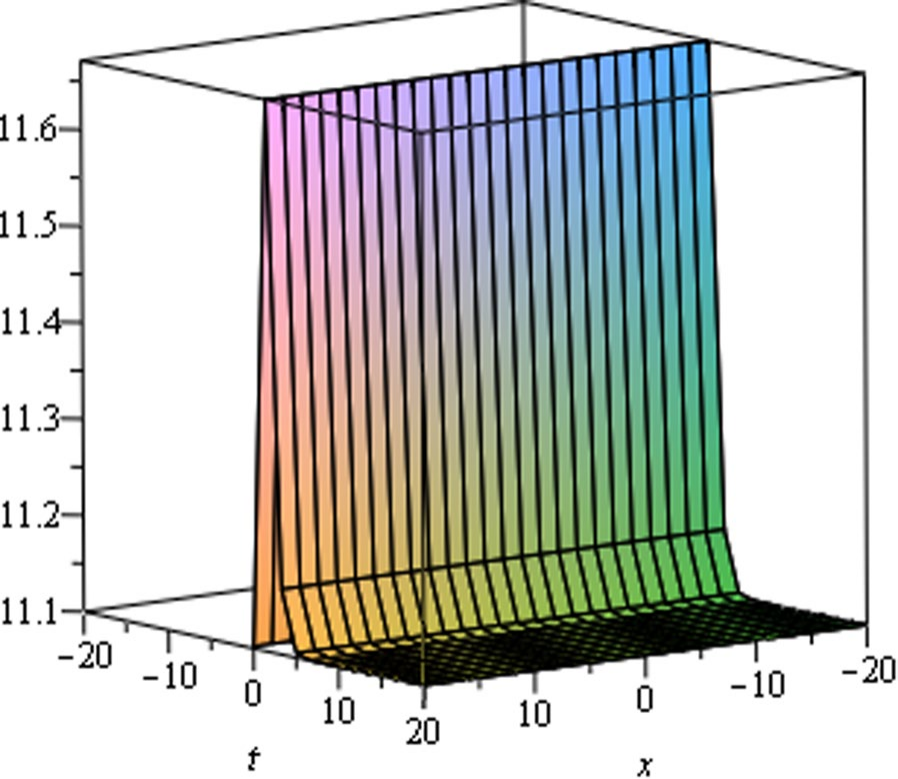
Singular solitary wave solution $u_1 \left({\eta}\right)$ when ${\text{a}}_{0} = 11.1, \mu = - 0.0002, {\text{x}} = 15, \alpha = 0.50.$

**Fig. 3:**
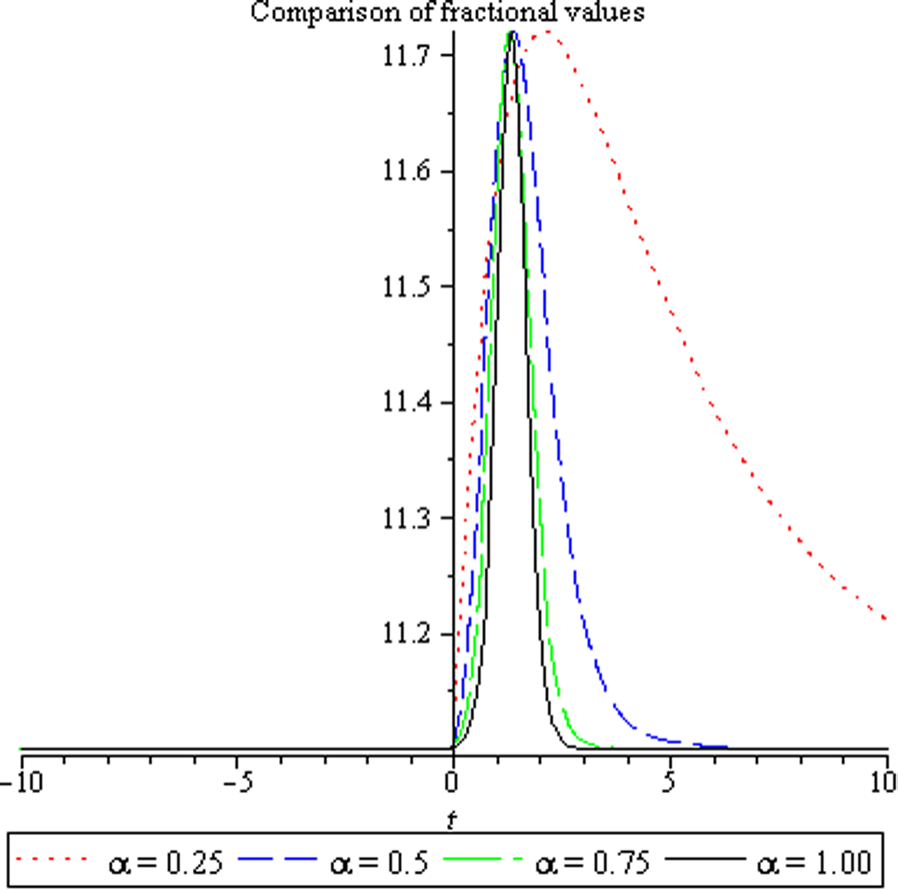
Comparison of solutions for different values of $\alpha$

**Fig. 4:**
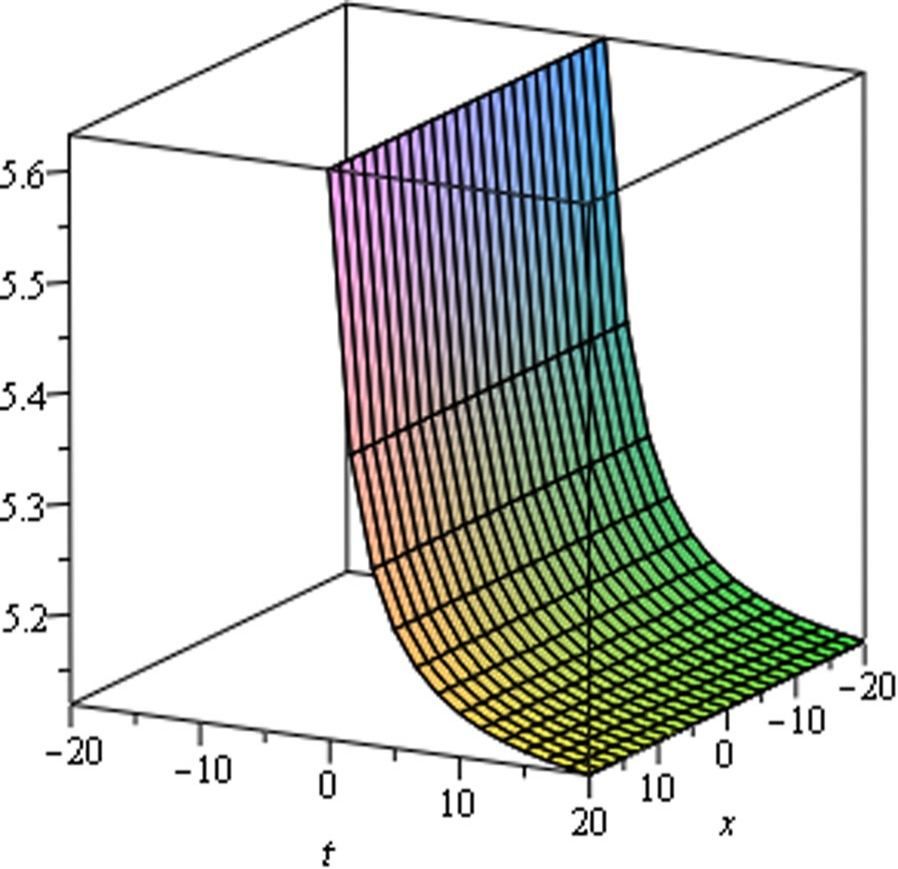
Singular Kink wave solution ${u}_{2} \left({\eta}\right)$ when ${a}_{0} = 5.1,\,{\mu}= 0.002,\, {x} = 2,\,{\alpha}= 0.50$

**Fig. 5:**
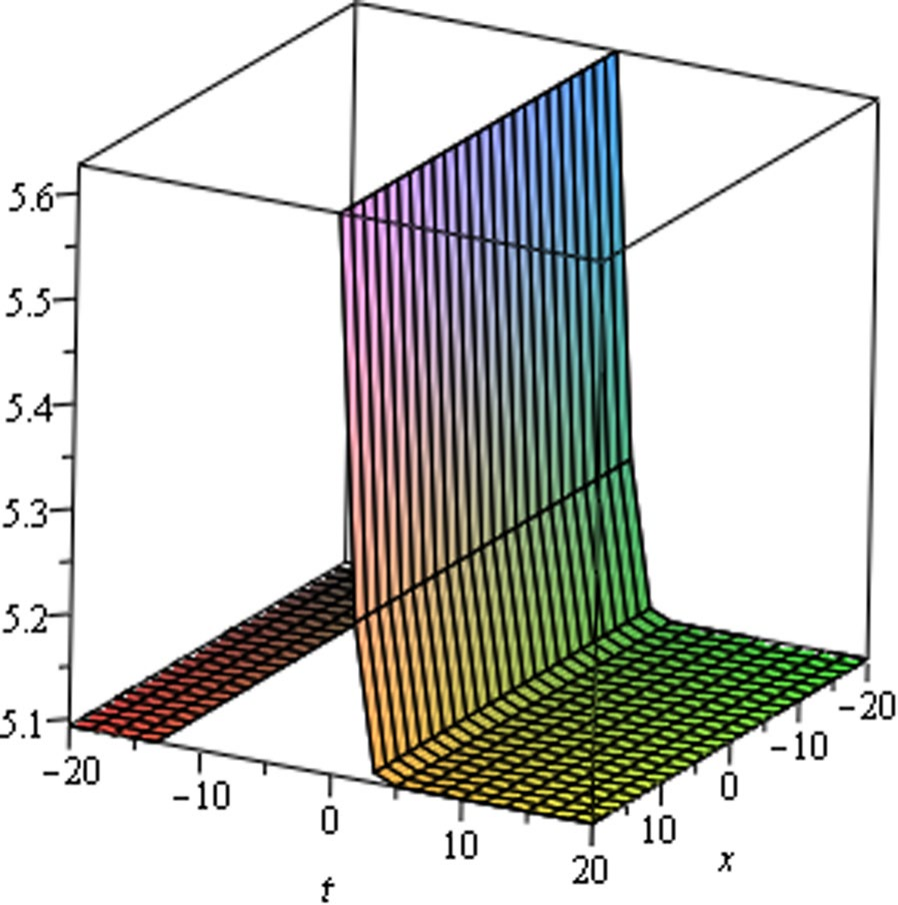
Singular Kink wave solution ${u}_{2} \left({\eta}\right)$ when ${a}_{0} = 5.1,\,{\mu}= 0.002,\, {x} = 2,\,{\alpha}= 0.75$

**Fig. 6:**
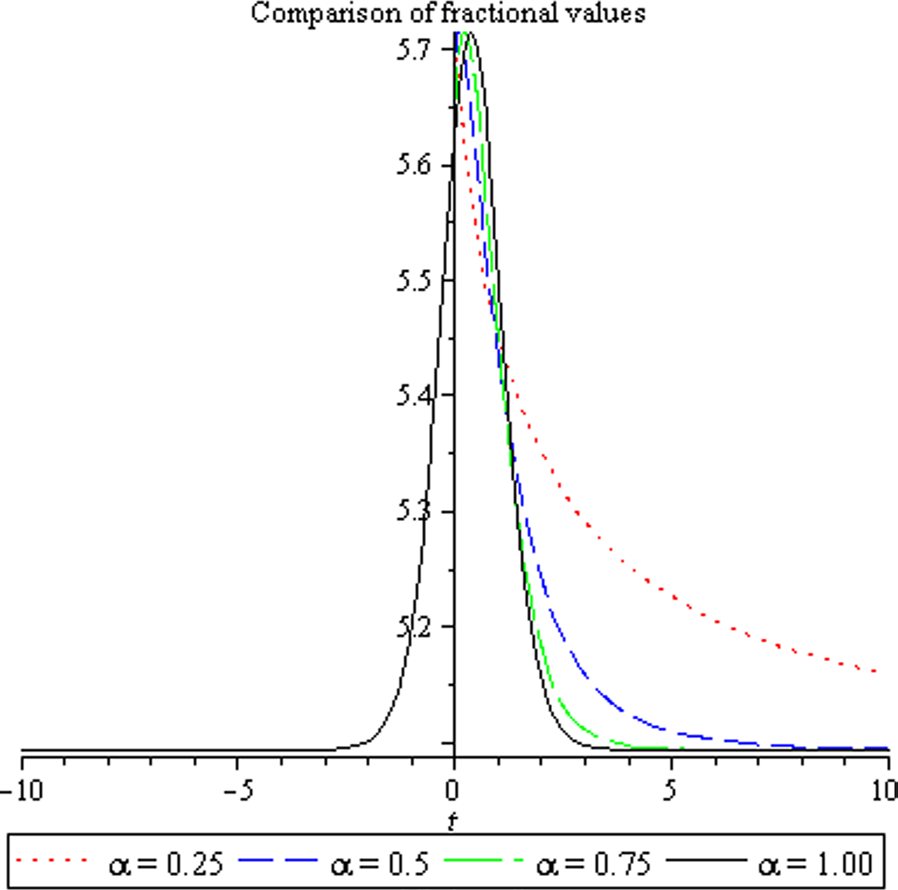
Comparison of solutions for different values of $\alpha$

**Fig. 7:**
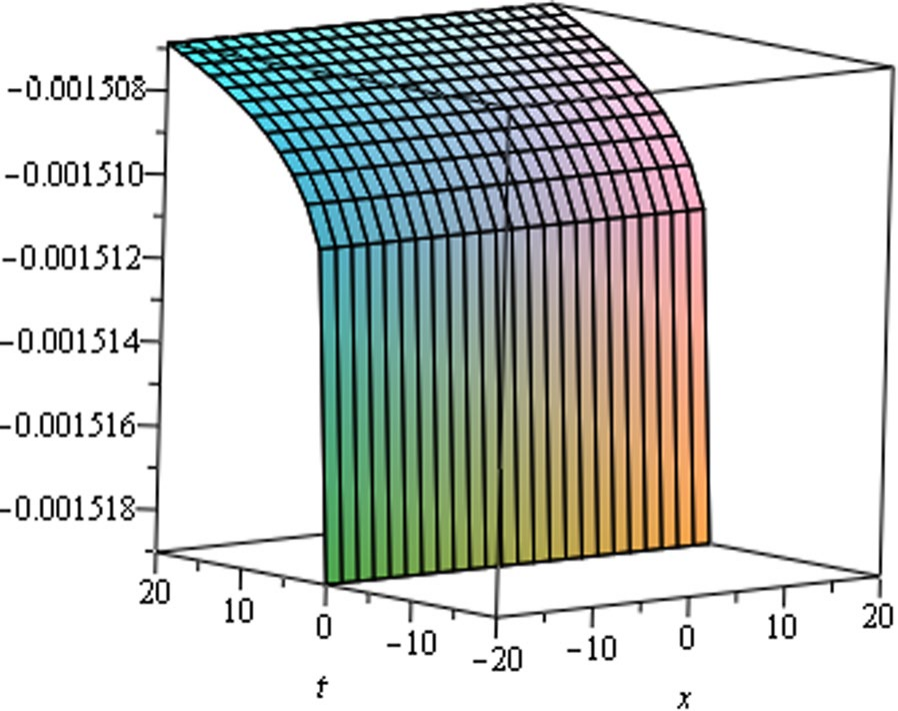
Singular Kink wave solution ${u}_{3} \left({\eta}\right)$ when ${a}_{0} = 0.0001,\,{\mu}= 0.001,\, {x} = 6,\,{\alpha}= 0.25$

**Fig. 8:**
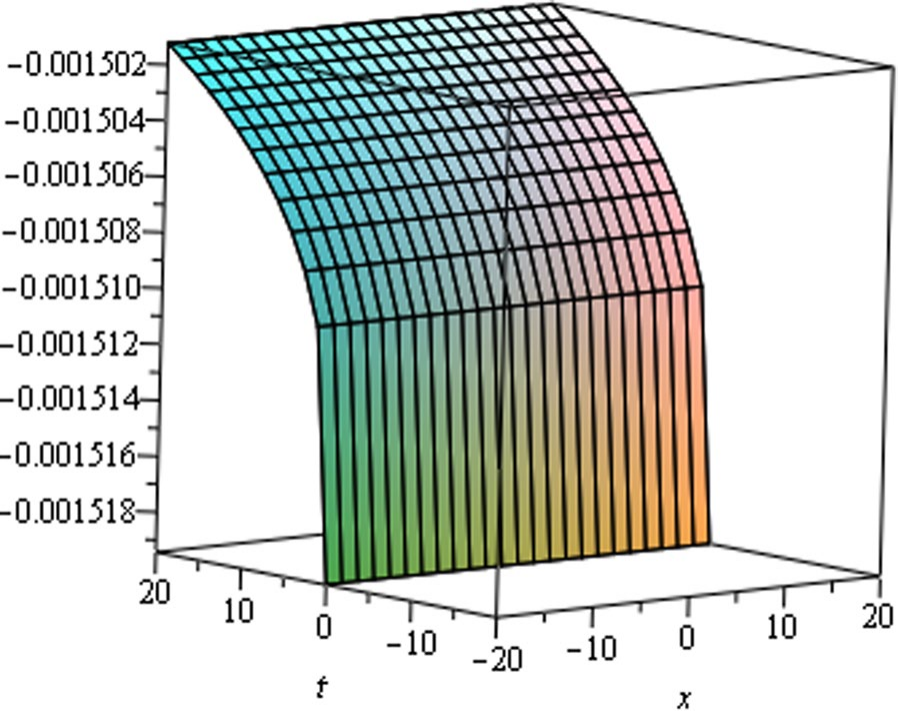
Singular Kink wave solution ${u}_{3} \left({\eta}\right)$ when ${a}_{0} = 0.0001,\,{\mu}= 0.001,\, {x} = 6,\,{\alpha}= 0.15$

**Fig. 9:**
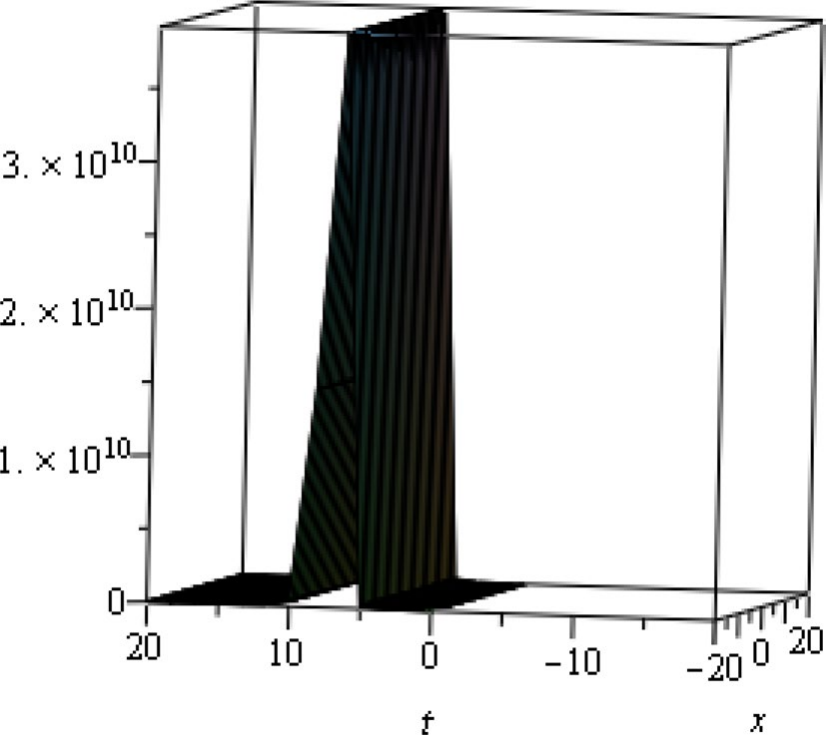
Singular solitary wave solution $u_{4}$ when ${a}_{0} = 0.01,\,{\mu}= 0.001,\, {x} = 0.1,\,{\alpha}= 0.50$

**Fig. 10:**
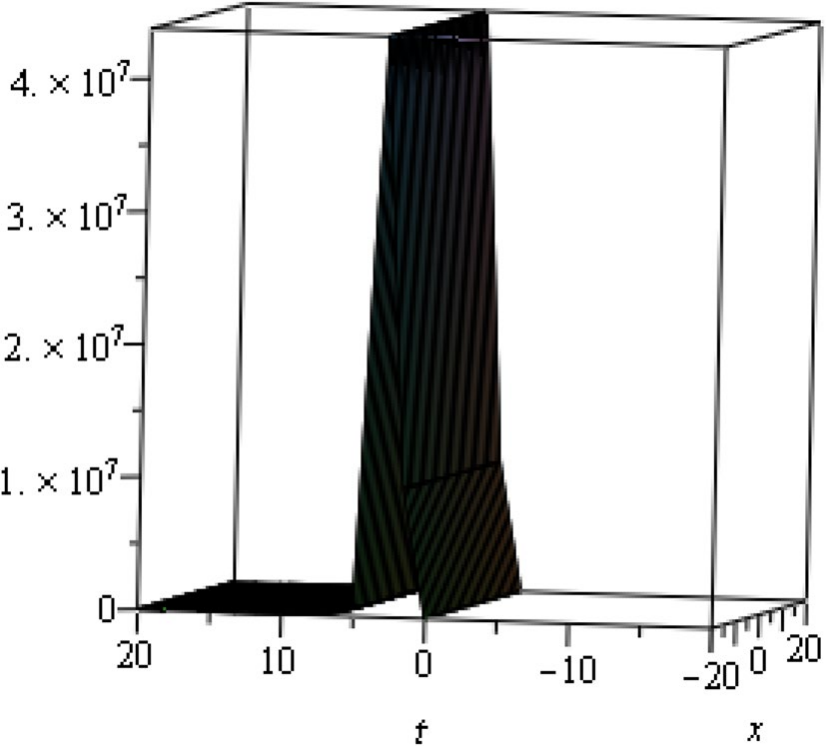
Singular solitary wave solution ${u}_{4}$ when ${a}_{0} = 0.01,\,{\mu}= 0.001,\, {x} = 0.1,\,{\alpha}= 0.25$

**Fig. 11:**
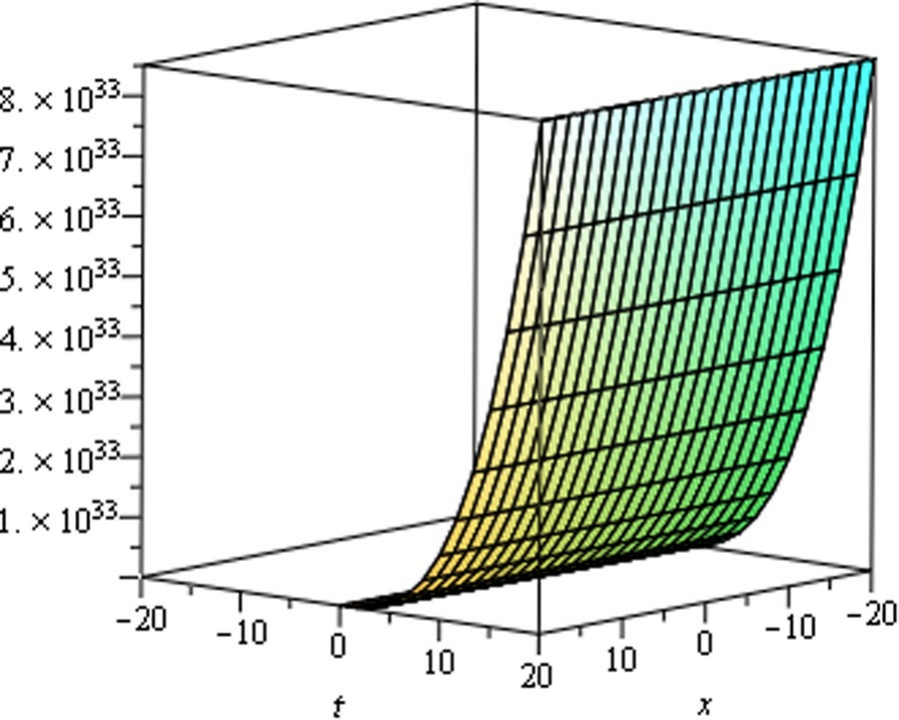
Singular Kink wave solution *u*_5_(*η*) when $a_{0} = - 2, \mu = 14, x = 18 \alpha = 1.00$

**Fig. 12:**
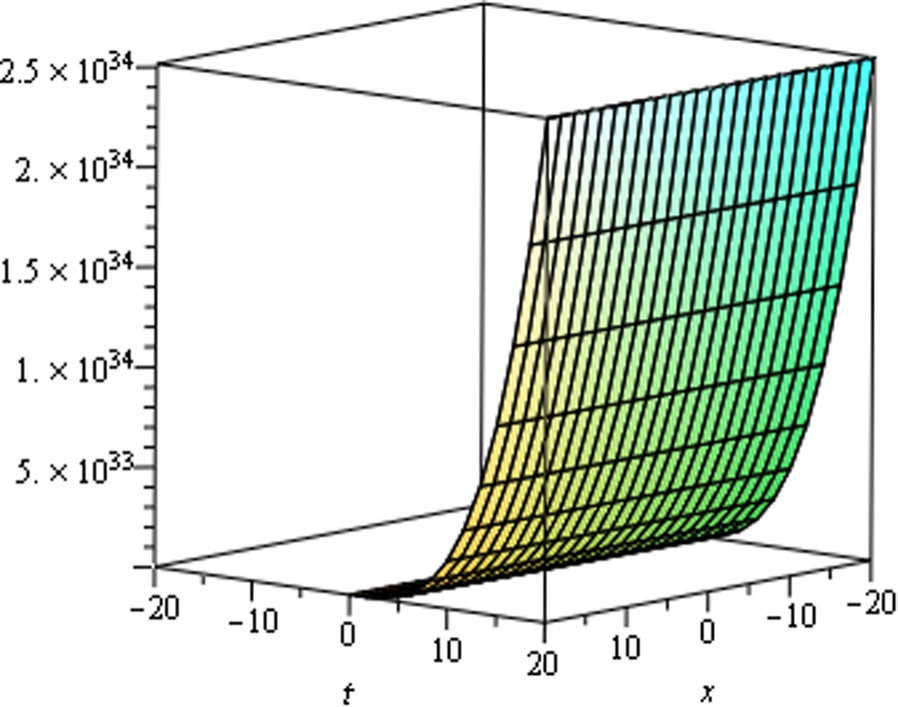
Singular Kink wave solution *u*_5_(*η*) when $a_{0} = - 2, \mu = 14, x = 18 \alpha = 0.75.$

The proposed method provides more general and abundant new solitary wave solutions with some free parameters. The traveling wave solutions have its extensive significance to interpret the inner structures of the natural phenomena. We have explained the different types of solitary wave solutions by setting the physical parameters as special values. In this paragraph, we will explain the physical elucidation of the solutions for the Kawahara equation for ${\mathbf{a}}_{{\mathbf{0}}} = {\mathbf{11}}{\mathbf{.1}},\; {\varvec{\upmu}} = -{\mathbf{0}}{\mathbf{.0002}},\; {\mathbf{x}} = {\mathbf{15}}, \;{\varvec{\upalpha}} = {\mathbf{0}}{\mathbf{.50}}, {\mathbf{u}}_{{\mathbf{1}}}$ shows the singular solitary wave solution as shown in Figs. 1, 2, 3). Figure 4 shows the shape of the singular kink wave solution of **u**_**2**_ for ${\mathbf{a}}_{{\mathbf{0}}} = {\mathbf{5}}{\mathbf{.1}},\; {\varvec{\upmu}} = {\mathbf{0}}{\mathbf{.002}}, \;{\mathbf{x}} = {\mathbf{2}}, \;{\varvec{\upalpha}} = {\mathbf{0}}{\mathbf{.75}}.$ Again singular Kink solution obtained in Fig. 5 of **u**_**2**_ for $\varvec{a}_{{\mathbf{0}}} = {\mathbf{5}}{\mathbf{.1}}, \;\varvec{\mu}= {\mathbf{0}}{\mathbf{.002}}, \;\varvec{x} = {\mathbf{2}}, \;{\varvec{\upalpha}} = {\mathbf{0}}{\mathbf{.50}}$ (Figs. 6, 7, 8, 9, 10, 11). Finally simple kink solution got from $\varvec{u}_{{\mathbf{5}}}$ for the choice of ${\mathbf{a}}_{{\mathbf{0}}} = - {\mathbf{2}}, \;{\varvec{\upmu}} = {\mathbf{14}}, \;{\mathbf{x}} = {\mathbf{18}}, {\varvec{\upalpha}} = {\mathbf{0}}{\mathbf{.75}}.$ which is shown in Fig. 12. In one asymptotic state to another asymptotic state, kink solitons are upsurge or descent. Such solitons are called topological solitons. The other exact solutions could be obtained from the remaining solution sets.

## Numerical discussion

We have obtained the exact solutions (29), (30) and (31) in the above study and to know the correctness we have matched those solutions with the exact solution (Bongsoo 2009).We note that the absolute errors given in the tables from the solutions we have obtained are very precise and accurate.

**Table 1:** Comparison of the absolute errors for the exact solution obtained by (29) with the exact solution (32) when

x\t	0.5	1	1.5	2	2.5
0.5	2.07927E−05	2.16050E−05	2.24665E−05	2.33804E−05	2.43496E−05
1.0	1.69635E−05	1.75796E−05	1.82331E−05	1.89262E−05	1.96615E−05
1.5	1.39182E−05	1.43854E−05	1.48811E−05	1.54068E−05	1.59644E−05
2.0	1.14683E−05	1.18227E−05	1.21985E−05	1.25972E−05	1.30201E−05
2.5	9.47090E−06	9.73956E−06	1.00245E−05	1.03269E−05	1.06475E−05

**Table 2:** Comparison of absolute errors obtained by (30) with the exact solution (32) when

x\t	0.5	1	1.5	2	2.5
0.5	2.80883E−05	2.69054E−05	2.57900E−05	2.47385E−05	2.37470E−05
1.0	3.40754E−05	3.25163E−05	3.10463E−05	2.96603E−05	2.83534E−05
1.5	4.21575E−05	4.01031E−05	3.81659E−05	3.63394E−05	3.46171E−05
2.0	5.29970E−05	5.02905E−05	4.77384E−05	4.53318E−05	4.30626E−05
2.5	6.74652E−05	6.39005E−05	6.05389E−05	5.73689E−05	5.43796E−05

**Table 3:** Comparison of absolute errors obtained by (31) with the exact solution (32) when

x\t	0.5	1	1.5	2	2.5
0.5	1.11802E−02	1.10649E−02	1.09508E−02	1.08381E−02	1.07266E−02
1.0	1.08009E−02	1.06829E−02	1.05663E−02	1.04512E−02	1.03374E−02
1.5	1.03542E−02	1.02348E−02	1.01170E−02	1.00008E−02	9.88610E−03
2.0	9.84771E−03	9.72834E−03	9.61069E−03	9.49476E−03	9.38053E−03
2.5	9.28994E−03	9.17198E−03	9.05588E−03	8.94165E−03	8.82927E−03

32 $$u\left( {x,t} \right) = \frac{420\beta }{164\alpha \gamma }\left[ {n\left( {x,t} \right)} \right]\left[ {\frac{{n\left( {x,t} \right)}}{2} + 1} \right]^{2}$$

where

33 $$n\left( {x,t} \right) = \frac{{1 - \left\{ {\cosh \left[ {\sqrt {\frac{\beta }{13\gamma } } \left( {x - \frac{{36\beta^{2} t}}{169\gamma } + m} \right)} \right] + \sqrt {p^{2} + 1} } \right\}}}{{\left\{ {\sinh \left[ {\sqrt {\frac{\beta }{13\gamma } } \left( {x - \frac{{36\beta^{2} t}}{169\gamma } + m} \right)} \right] + p} \right\}}}$$

## Conclusion

With the help of a suitable transformation and the $\exp \,\left( { - \varphi \left( \eta \right)} \right)$-expansion method, we obtained different types of exact solutions for fractional Kawahara equation. The obtained results show that the proposed technique is effective and capable for solving nonlinear fractional partial differential equations. In this research, some exact solitary wave solutions, mostly solitons and kinks solutions are obtained through the hyperbolic, trigonometric, exponential and rational functions. It is observed that the proposed method fully validate the competence and reliability of computational work as evident from Tables 1, 2 and 3 and may be utilized for other physical problems.
